# Diagnostic Dilemma: Adult Onset Still's Disease Mimicking Lymphoma—A Case Report and Literature Review

**DOI:** 10.1155/crii/5533371

**Published:** 2025-08-08

**Authors:** Mohannad N. AbuHaweeleh, Al-jouhara Albaloshi, Mohammed Al-Hor, Majed Al-Theyab, Ahmad Almaslamani, Basant Elsayed, Moaz O. Moursi, Abdelrahman Hamad

**Affiliations:** ^1^College of Medicine, Qatar University, Doha, Qatar; ^2^Department of Internal Medicine, Hamad Medical Corporation, Doha, Qatar

**Keywords:** adult onset Still's disease, case report, fever of unknown origin, lymphadenopathy, lymphoma

## Abstract

**Introduction:** Adult-onset Still's disease (AOSD) is a rare systemic inflammatory condition with hallmark features of spiking fevers, arthritis, and a salmon-colored maculopapular rash. It typically affects young adults, with a bimodal age distribution of 15–25 and 36–46 years. The prevalence of AOSD ranges from 1 to 34 cases per million people, with an incidence rate of 0.16–0.4 per 100,000 individuals. AOSD's etiology remains unclear but is thought to involve genetic and environmental factors. Diagnosis relies on clinical criteria, such as the Yamaguchi criteria, and exclusion of other conditions. Misdiagnosis is common, particularly in regions where infections and malignancies with overlapping features are prevalent. This case report highlights a diagnostically challenging case of AOSD in a young woman, emphasizing the importance of thorough evaluation and accurate diagnosis.

**Case Presentation:** A 21-year-old female with no prior comorbidities presented with persistent fever, a transient salmon-colored rash, and polyarthritis involving the wrists, shoulders, ankles, and small joints. Three months prior, she had been treated for left submandibular sialadenitis attributed to mumps. Laboratory work revealed elevated C-reactive protein (CRP), ferritin, and LDH levels with a normal erythrocyte sedimentation rate (ESR) and autoimmune profile. Imaging studies, including PET-CT, suggested malignancy, raising suspicion of lymphoma. However, lymph node biopsy showed reactive hyperplasia without evidence of malignancy. Ultimately, a diagnosis of AOSD was established based on clinical and laboratory findings. The patient was initiated on anakinra and prednisolone, leading to significant improvement. At a 2-month follow-up, she had complete resolution of symptoms.

**Conclusion:** AOSD presents a significant diagnostic challenge due to its rarity and symptom overlap with infections, malignancies, and autoimmune diseases. This case highlights the importance of a thorough clinical evaluation and the application of established diagnostic criteria to facilitate early diagnosis and timely management.

## 1. Introduction

Adult-onset Still's disease (AOSD) is a rare and systemic autoinflammatory disorder with key clinical features including high-spiking fevers, arthritis, and a distinctive salmon-colored maculopapular rash [1]. AOSD primarily affects young adults, with a bimodal age distribution, most commonly presenting in two age groups: 15–25 and 36–46 years [2]. The current data suggests that the incidence of AOSD is 0.16 and 0.4 per 100,000 people [3] and the prevalence rate is between 1 and 34 cases per 1 million people [4]. The exact etiology of AOSD remains poorly understood [5]; however, it is postulated that both genetic susceptibility and environmental or infectious triggers may contribute to the onset of the disease [6].

Genetic factors, including specific human leukocyte antigen (HLA) alleles, may predispose individuals to developing AOSD, though no single causative factor has been definitively identified. Laboratory findings typically reveal markedly elevated inflammatory markers, such as C-reactive protein (CRP), erythrocyte sedimentation rate (ESR), and ferritin levels. However, when AOSD is complicated by macrophage activation syndrome (MAS), ESR may paradoxically decrease which can be attributed to the consumption of fibrinogen and other coagulation factors during the hyperinflammatory state of MAS, leading to hypofibrinogenemia [7]. The management of AOSD primarily focuses on mitigating the systemic inflammatory response and preventing organ damage by administering high-dose corticosteroids or conventional DMARDs like methotrexate [8]. In refractory/complex cases, biologic agents such as interleukin-1 (IL-1) and interleukin-6 (IL-6) inhibitors have been used with varying success [2, 9].

Due to its rarity and the overlap of its symptoms with other conditions, AOSD is often misdiagnosed or diagnosed late [10]. This diagnostic delay can lead to complications such as joint damage, systemic involvement, and increased morbidity [11]. A study reported a misdiagnosed AOSD as septic arthritis [12], which can be life threatening if not adequately treated. Additionally, AOSD can be easily misdiagnosed clinically as infectious lesions or other autoimmune diseases, such as systemic lupus erythematosus, because of overlapping symptoms [13]. A comprehensive review emphasized the need for further studies to clarify the disease's pathogenesis, clinical features, and treatment outcome [14].

This case report describes a young female diagnosed with AOSD after undergoing a series of inconclusive investigations, highlighting the diagnostic challenges associated with AOSD.

## 2. Case Presentation

A 21-year-old woman with no known comorbidities presented to the emergency department with a 1-month history of persistent fever and rash. Three months earlier, she had been hospitalized for left neck swelling and was diagnosed with left submandibular sialadenitis accompanied by ipsilateral submandibular and cervical lymphadenopathy, attributed to a mumps infection.

At the time of the current presentation, she reported a daily fever at home, typically ranging from 38 to 39°C, occurring throughout the day without a specific pattern and alleviated by paracetamol. The patient reported an intermittent, nonpruritic, pink rash predominantly affecting her arms and legs. The rash appeared mainly at night and faded by morning. On examination, it was warm to the touch but nonblanching. She also experienced joint pain predominantly in the wrists, shoulders, ankles, and small joints of the hands and feet, though without any associated swelling or warmth. Additional symptoms included generalized fatigue and occasional mild headaches. The left neck swelling, noted since the mumps infection, remained stable. She denied respiratory, abdominal, or urinary symptoms and reported no night sweats, weight loss, oral, or genital ulcers.

She had no significant past medical or surgical history apart from the recent hospitalization mentioned above, and she was not on any regular medications. She is allergic to ceftriaxone and clindamycin. There was no notable family history of relevance. She is a college student studying information technology, a nonsmoker, and has no history of alcohol or drug use. She reported no recent travel history. She was up to date with all age-appropriate immunizations, including the MMR vaccine.

On examination, her temperature was 36.9°C, pulse 77 beats per minute, respiratory rate 17 breaths per minute, blood pressure 109/73 mmHg, and oxygen saturation was 97% on room air. Her BMI was 24 kg/m^2^. She appeared generally well and was sitting comfortably in bed. A blanchable, pink maculopapular rash was noted on exposed body parts, with no mucosal involvement. Lymph node examination revealed firm, nodular, mobile, nontender, nonerythematous lymphadenopathy in the left cervical, submandibular, and axillary regions. The remainder of the systemic examination, including pulmonary, cardiovascular, and abdominal systems, was unremarkable. No lower limb edema or signs of joint arthritis were observed.

She was admitted under the general internal medicine department as a case of possible mumps reactivation, with differential diagnoses, including AOSD, Kikuchi-Fujimoto disease, autoimmune disease, and malignancy.

## 3. Methods

### 3.1. Investigations

Blood work (Table 1) revealed a normal complete blood count, except for mild microcytic anemia, and a normal coagulation profile. Kidney, liver, and thyroid function tests were all within reference ranges. Significant findings included elevated lactate dehydrogenase (LDH), mildly elevated CRP, and normal ESR. Ferritin levels were elevated, with the remaining iron profile within normal limits. Persistently positive mumps serology (IgM and IgG), consistent with the recent mumps infection three months prior, was noted, although mumps PCR testing was negative. Other notable negative results included an autoimmune profile, Quantiferon, HIV, hepatitis serologies, rubella, measles, toxoplasma, *Treponema pallidum*, and parvovirus B19 serologies. Blood and urine cultures also returned negative results. Lymphocyte subset analysis was normal, and immunoglobulin levels showed no hypogammaglobulinemia. Serum protein electrophoresis (SPE) revealed an inflammatory pattern, characterized by increased alpha and gamma globulins, with no monoclonal protein detected.

Neck ultrasound, compared to the previous study, demonstrated a mild interval decrease in the size of the left submandibular lymphadenopathy. The left submandibular gland remained minimally heterogeneous with interval size reduction. No evidence of collection or active sialadenitis was noted. Chest x-ray was unremarkable.

**Figure 1:**
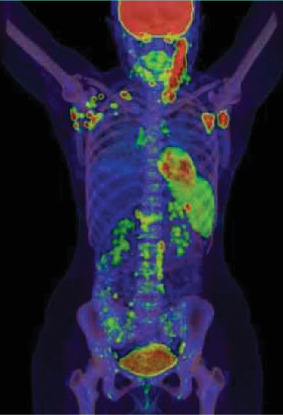
A coronal whole-body PET-CT demonstrating multiple areas of increased metabolic activity. There is intense FDG uptake involving lymph nodes both above and below the diaphragm, consistent with widespread nodal involvement. Prominent heterogeneous FDG uptake is observed in the bone marrow, which may suggest marrow activation or infiltration. Additionally, there is an intensely hypermetabolic focus located immediately posterior to the right kidney.

As part of the ongoing diagnostic workup, it was decided to proceed with a PET-CT scan for further evaluation (Figure 1). PET-CT scan findings suggested a malignant process involving lymph nodes above and below the diaphragm, with an imaging pattern highly indicative of lymphoma. Splenic infiltration was considered likely if lymphoma was confirmed. The scan also showed prominently increased heterogeneous bone marrow uptake, prompting a recommendation for bone marrow aspiration if malignancy was proven. Additionally, an intensely hypermetabolic focus posterior to the right kidney raised suspicion of malignancy; however, suboptimal anatomical registration left uncertainty regarding its origin (renal or extra-renal), warranting further evaluation with ultrasound or contrast-enhanced CT for better delineation. Enlarged left axillary lymph nodes measuring 2.4 cm were identified as accessible for sampling.

### 3.2. Intervention

A left axillary lymph node excisional biopsy revealed an enlarged lymph node with atypical paracortical hyperplasia, showing up to 80% proliferation fraction with atypical cells, alongside background reactive lymphoid follicular hyperplasia and minimal plasmacytosis. Although these findings could be associated with reactive conditions, early T-cell neoplasia could not be entirely excluded. Epstein–Barr virus encoded RNA (EBER) in situ hybridization was negative. PCR analysis showed Gaussian distributions consistent with polyclonal T and B cells. Flow cytometry on lymph node showed approximately 46% T-cells, 42% B-cells, and 2% plasma cells with no definitive immunophenotypic evidence of monotypic B-cell population or monotypic plasma cell population. Molecular analysis via PCR for T-cell receptor (gamma and beta) and immunoglobulin heavy chain gene rearrangements revealed Gaussian distributions consistent with polyclonal T- and B-cell populations, strongly supporting a diagnosis of reactive lymphoid hyperplasia rather than lymphoma. Tuberculosis workup and cultures from the biopsy were negative.

The patient was referred to the lymphoma multidisciplinary team (MDT), which recommended completing the workup with a bone marrow examination, obtaining rheumatology input for the possibility of AOSD, and close observation, with consideration for a repeat PET-CT scan if symptoms persist.

The patient's family decided to seek medical care abroad and traveled to USA for further evaluation and management. A bone marrow biopsy conducted there revealed no abnormalities. After thorough assessment, she was diagnosed with AOSD based on her clinical presentation and diagnostic findings. Treatment with Anakinra and Prednisolone was initiated, resulting in significant clinical improvement. At 2-month follow up, the patient experienced complete resolution of fever, rash, and joint pain, along with a marked reduction in neck swelling.

## 4. Discussion

This case report details the diagnosis of AOSD in a young female following a series of inconclusive tests, emphasizing the diagnostic difficulties encountered with AOSD.

A fever with lymphadenopathy of unknown origin is challenging to assess owing to the extensive differential diagnosis, including infections (such as viral infections and tuberculosis), malignancies (including lymphomas), and autoimmune illnesses (like SLE). Many variables help in identifying the cause of such presentation, including demographics, such as tuberculosis-endemic regions, in which granulomatous lymphadenitis from *Mycobacterium tuberculosis* is prevalent [15]. Also, laboratory tests such as positive autoimmune work up as in SLE or positive lymph node biopsy and bone marrow diagnosing lymphoma. Additionally, some clinical and laboratory distinctive findings together may point to a specific cause such as salmon-colored rash, recurrent fevers, and markedly elevated serum ferritin levels can be seen in ASOD. However, many patients may present with incomplete or overlapping picture that necessities further diagnostic tools to reach to a diagnosis such as imaging and lymph node biopsy [16].

The Yamaguchi criteria, commonly used for the diagnosis of AOSD, require the presence of at least five features—of which at least two must be major—including high-grade fever, arthralgia or arthritis, a typical salmon-pink rash, and leukocytosis as major criteria, and sore throat, lymphadenopathy, hepatosplenomegaly, elevated liver enzymes, and negative ANA and RF as minor criteria [17]. The various presentations of AOSD emphasis the challenge of diagnosing patients with AOSD and explain the delay in identifying such cases. Although fever and salmon-colored rashes are hallmark features, nocturnal rash fading by morning, as observed in our patient, is less frequently reported. In a systematic review of AOSD cases, diurnal variation of rash was noted in 51% of cases, highlighting its relative rarity [18]. Additionally, vesicular eruptions overlying a morbilliform rash, as seen in this case, deviate from classical dermatological findings, which are typically maculopapular or erythematous. In AOSD, laboratory data commonly demonstrate neutrophilic leukocytosis, elevated liver transaminases, and dramatically increased serum ferritin levels [19]. In our case, lab results were not conclusive, and they only showed hyperferritinemia. However, other nonspecific findings like elevated LDH were present.

AOSD frequently mimics hematologic malignancies due to shared clinical features, including fever, lymphadenopathy, and systemic inflammation. PET-CT findings in AOSD can show intense FDG uptake in lymph nodes, spleen, and bone marrow, often leading to misinterpretation as lymphoma. Conversely, lymphoma presents with different organ involvement and reduced patterns of hypermetabolism in some tissues [20]. Incorporating PET-CT features with clinical and laboratory data enables the development of diagnostic models, enhancing accuracy and aiding clinicians in making more informed decisions, ultimately facilitating timely and appropriate treatment [20]. However, histopathological confirmation remains the gold standard for differentiating between the two conditions. As in our case, PET-CT findings suggested a malignant process, with hypermetabolic lymphadenopathy above and below the diaphragm. However, histopathological examination of an excised lymph node revealed reactive hyperplasia with no evidence of malignancy, shifting the diagnostic focus towards inflammatory or autoimmune etiologies. Similarly, prominent lymphadenopathy with PET scan evidence of malignancy mimicking lymphoma has been reported in isolated cases [21], emphasizing the importance of histopathological differentiation. Our patient's negative lymph node biopsy initially challenged the diagnosis, underscoring the diagnostic dilemma in cases of lymphadenopathy with systemic features. While Kikuchi–Fujimoto disease was considered, the absence of necrotizing lymphadenitis excluded this diagnosis. Furthermore, suspected infectious triggers like EBV or mumps can be potential inciting events for AOSD flares [22].

For diagnosis, the Yamaguchi criteria are commonly employed, where a minimum of five criteria must be fulfilled, including at least two major criteria, with any diagnosis excluded in the presence of any of the exclusion criteria. Methylprednisolone pulses are used in severe situations where rapid control of inflammation is necessary. Disease-modifying antirheumatic drugs (DMARDs), such as methotrexate, are given to patients with recurrent symptoms or who need long-term steroid treatment. Therefore, it is important to recognize AOSD early and treat effectively, in order to prevent complications and improve patient outcomes [11, 23].

## 5. Conclusion

In conclusion, this case illustrates the diagnostic complexity of AOSD and its potential to mimic hematologic malignancies. A structured diagnostic approach, incorporating clinical assessment, imaging, and histopathological confirmation, is essential for timely and accurate diagnosis. Early recognition and targeted treatment can significantly improve patient outcomes.

## Figures and Tables

**Table 1:** Blood investigations.

Lab test	Result	Unit	Reference range
WBC	8	×10^3^/μL	4–10
HB	11.4	g/dL	12–15 mg/dL
MCV	79.4	—	83–101
Platelets	245	×10^3^/μL	150–400
PT	12.5	Seconds	9.7–11.8
INR	1.1	—	—
Creatinine	46	μmol/L	44–80
Urea	2.4	mmol/L	2.5–7.8
Sodium	138	mmol/L	133–146
Potassium	4.4	mmol/L	3.5–5.3
Bicarbonate	22	mmol/L	22–29
Albumin	35	g/L	35–50
Total protein	77	g/L	60–80
Total bilirubin	5	μmol/L	0–21
ALT	21	U/L	0–33
AST	17	U/L	0–32
ALP	63	U/L	35–104
GGT	8	U/L	6–42
CRP	11.9	mg/L	0–5
ESR	29	mm/h	2–37
Lactate dehydrogenase	300	U/L	135–214
Ferritin	542	μg/L	12–114
Iron	20	μmol/L	6–35
TIBC	58	μmol/L	45–80
Transferrin	34	g/L	2–3.6
Fe% saturation	13%	—	15–45
Thyroid stimulating hormone	2.97	mIU/L	0.3–4.2
Hepatitis B surface antigen	Negative	—	—
Hepatitis B surface antibody	Negative	—	—
Hepatitis C antibody	Negative	—	—
Quantiferon TB gold plus	Negative	—	—
Antinuclear antibody (CTD screen)	Negative	—	—
Antineutrophil cytoplasmic antibodies	Negative	—	—
Antidouble stranded DNA antibody	Negative	—	—
Rheumatoid factor	Negative	—	—
AntiCCP antibody	Negative	—	—
Brucella IgG antibody	Negative	—	—
Brucella IgM antibody	Negative	—	—
*Treponema pallidum* antibody	Negative	—	—
HIV antigen/antibody combo	Negative	—	—
EBV capsid antigen IgG	Positive	—	—
EBV capsid antigen IgM	Negative	—	—
Measles IgG antibody	Positive	—	—
Measles IgM antibody	Negative	—	—
Parvovirus B12 IgG antibody	Negative	—	—
Parvovirus B12 IgM antibody	Negative	—	—
Rubella IgG antibody	Positive	—	—
Rubella IgM antibody	Negative	—	—
Toxoplasma IgG antibody	Negative	—	—
Toxoplasma IgM antibody	Negative	—	—
Mumps IgG antibody	Positive	—	—
Mumps IgM antibody	Positive	—	—
Mumps virus PCR	Negative	—	—
Parcho virus PCR	Negative	—	—
Enterovirus PCR	Negative	—	—
Cytomegalovirus (CMV) PCR	Negative	—	—
Epstein–barr virus (EBV) PCR	Negative	—	—
Varicella zoster virus PCR	Negative	—	—
Herpes simplex virus (HSV 1) PCR	Negative	—	—
Herpes simplex virus (HSV 2) PCR	Negative	—	—

Abbreviations: ALT, alanine transaminase; Anti-CCP, anticyclic citrullinated peptide; AST, aspartate transaminase; CR, creatinine; CRP, C-reactive protein; ESR, erythrocyte sedimentation rate; GGT, gamma-glutamyl transferase; HB, hemoglobin; HIV, human immunodeficiency virus; INR, international normalized ratio; MCV, mean corpuscular volume; PT, prothrombin time; WBC, white blood cell count.

## Data Availability

Data sharing is not applicable to this article as no datasets were generated or analyzed during the current study.
